# Assessment of Japanese Stimulant Control Law Offenders Using the Addiction Severity Index—Japanese Version: Comparison with Patients in Treatment Settings

**DOI:** 10.3390/ijerph6123056

**Published:** 2009-12-03

**Authors:** Takashi Watanabe, Yasukazu Ogai, Takehiro Koga, Eiichi Senoo, Kazuhiko Nakamura, Norio Mori, Kazutaka Ikeda

**Affiliations:** 1 Hamamatsu University School of Medicine, 1-20-1 Handayama, Higashi-ku, Hamamatsu, Japan; E-Mails: takashi_matuki@yahoo.co.jp (T.W.); nakamura@hama-med.ac.jp (K.N.); morin@hama-med.ac.jp (N.M.); 2 Shizuoka Prison, 3-1-1 Higashichiyoda, Aoi-ku, Shizuoka, Japan; E-Mail: kg-ronnie@fmwbs.jp; 3 Division of Psychobiology, Tokyo Institute of Psychiatry, 2-1-8 Kamikitazawa, Setagaya-ku, Tokyo 156-8585, Japan; E-Mail: y-oogai@prit.go.jp; 4 Division of Social Psychiatry, Tokyo Institute of Psychiatry, 2-1-8 Kamikitazawa, Setagaya-ku, Tokyo 156-8585, Japan; E-Mail: senoo@prit.go.jp

**Keywords:** methamphetamine, Addiction Severity Index, Japanese, prison, correctional facilities

## Abstract

The present study assessed problems in Japanese prisoners (inmates) who abused methamphetamine. Fifty-two male inmates were assessed in 2005–2007 using the Addiction Severity Index-Japanese version and compared with 55 male methamphetamine abusers in hospitals and recovery centers. The *χ*^2^ and Mann-Whitney-Wilcoxon tests showed that the inmates had a significantly lower education level, more frequently had full-time jobs, had more experience living with a sexual partner, and more frequently had a history of juvenile delinquency and criminal records than patients. Although psychiatric symptoms, such as depression, anxiety, and hallucinations, were not common among inmates, suicidal behavior and trouble controlling violence were common in both groups.

## 1. Introduction

The production, trafficking, and use of amphetamine-type stimulants have increased significantly since the 1990s throughout East Asia and the Pacific countries [1]. The most prevalent amphetamine-type stimulant in East Asia is methamphetamine, the principal drug involved in drug abuse cases in Japan [2,3]. Stimulant dependence presents a serious problem, not only for patients, but also for Japanese society [4]. For example, about 25% of convicted prisoners committed offenses under the Stimulant Control Law [5]. Although many Japanese methamphetamine abusers have received only punishment rather than medical treatment [3], drug-abuse problems in inmates have not been sufficiently investigated. Moreover, appropriate assessment and treatment of substance abuse have not been provided in Japanese prisons, partly because the number of inmates in Japan exceeds prison capacity.

In a study conducted at a Japanese hospital, methamphetamine abusers with serious criminal records tended to administer the drug by injection and had less chronic psychosis than those with less serious criminal records [6]. In a study of adolescents at a juvenile detention home, Miura *et al*. [7] reported that gender (female), age, number of admissions, violence, history of psychiatric treatment, and family history of drug misuse were significantly associated with methamphetamine use. Matsumoto *et al.* [8] reported no significant correlation between drug abuse and antisocial behaviors in male juvenile delinquents, although alcohol abuse has been hypothesized to promote these behaviors. The aforementioned studies, however, did not include Japanese inmates under the Stimulant Control Law in their sample. Although Matsumoto *et al.* [9] found a relationship between childhood tendencies toward attention-deficit/hyperactivity disorder and illicit drug abuse in Japanese prisoners, they did not compare the characteristics of methamphetamine abusers in prison with those in treatment facilities. Investigation of methamphetamine abusers in correctional settings and comparison of the results with those in treatment settings would elucidate the specific characteristics of methamphetamine abusers in correctional settings.

The Addiction Severity Index (ASI) [10] is used worldwide and is one of the few standardized instruments that address drug abuse. The ASI assesses legal status in addition to medical status, employment/support status, drug use status, alcohol use status, family/social status, and psychiatric status. These features make the ASI particularly useful for practitioners working with substance-abusing offenders [11]. For example, some studies used the ASI with inmates to confirm their common characteristics [12], to compare inmates with other drug abusers [10], and to confirm the effectiveness of the ASI as a screening tool for substance use disorders [13]. The ASI was also used to examine associations between psychosocial and criminal factors [14], relationships between prescription drug abuse and addiction severity [15], gender differences in substance abuse disorders [16], the effectiveness of methadone maintenance treatment [17], and the predictors of treatment motivation [18]. The ASI-Japanese version (ASI-J) has acceptable reliability and validity for Japanese drug abusers in treatment settings [19].

Using the ASI-J, the present study assessed the characteristics and problems of Japanese inmates who abuse methamphetamine by comparing them with methamphetamine-abusing patients in treatment settings. Revealing such characteristics and problems may contribute to improving substance abuse treatment policy and improving the effectiveness of interventions designed to reduce stimulant abuse and its adverse consequences.

## 2. Methods

### 2.1. Recruitment Site

The participants were recruited from an adult male-only prison in Shizuoka, Japan, from September 2005 to July 2006 and in February 2007. In this prison, offenders mainly came from Tokyo or Shizuoka prefectures. In cases of repeat offenders, the inmates were detained in this prison for the first three months of their sentence. In October 2005, for example, 178 (164 Japanese and 14 foreigners) of the 1,407 total inmates were imprisoned for methamphetamine use as a result of the Stimulant Control Law.

### 2.2. Participants and Procedure

The study was approved by the Institutional Review Board of each facility. Sixty-three Japanese adult (>20 years old) male inmates who were arrested for methamphetamine use based on the Stimulant Control Law were sequentially approached to participate in this study. They were recruited and interviewed within three months after their imprisonment. Of the 63 inmates, five (7.9%) refused to participate, and the remaining 58 (92.1%) were administered the Structured Clinical Interview of the *Diagnostic and Statistical Manual of Mental Disorders*, 4th edition (DSM-IV) [20], Substance Abuse Disorders module. After the interview, five (7.9%) inmates were excluded from the study because they were not diagnosed with substance abuse or dependence. Written informed consent was obtained from the remaining 53 (84.0%) inmates. No inmates presented with acute psychosis.

The ASI-J was administered to the 53 inmates to assess their characteristics and the severity of their problems. They were asked about their status before they had been arrested by the police. One inmate was excluded because of a pathologically disorganized response. Thus, data from 52 (82.5%) inmates were included in the statistical analysis.

One psychiatrist and two clinical psychologists recruited participants and interviewed them. To achieve inter-rater reliability, two interviewers received six sessions of training interviews with another expert interviewer to confirm rating consensus between all interviewers. Each interview for each participant took approximately 60 min to complete.

### 2.3. Comparison with Participants in Treatment Settings: Recruitment Criteria and Facilities

The ASI-J was administered to 111 drug abusers in treatment settings to examine its reliability and validity [19]. These participants were recruited at three hospitals and two recovery facilities between January 2002 and September 2004. Of the 111 drug abusers, 55 were included in the present study using the following criteria: at least 18 years old, male Japanese with a history of methamphetamine abuse, diagnosed as a drug abuser or drug dependent based on DSM-IV criteria, and an inpatient or outpatient at a Japanese mental hospital or recovery center or a person who was recovering from stimulant abuse and working in a recovery center. Patients in the acute phase of psychosis were excluded.

The numbers of participants from each facility were 23 (six inpatients, 17 outpatients, 0 recovering) from Tokyo Metropolitan Matsuzawa Hospital in Tokyo, 10 (one inpatient, nine outpatients, 0 recovering) from the National Center of Neurology and Psychiatry Musashi Hospital in Kodaira, two (one inpatient, one outpatient, 0 recovering) from Fukko-kai Tarumi Hospital in Kobe, 17 (eight inpatients, three outpatients, six recovering) from Self Support Services in Tokyo, and three (three inpatients, 0 outpatient, 0 recovering) from GAIA in Naha. Inpatients were asked about their status during the 30 days prior to their admission to the substance treatment facility. Outpatients and recovering patients were simply asked about their status during the 30 days prior to the ASI interview.

### 2.4. Statistical Analysis

Significant differences between two ASI datasets were examined using the *χ^2^* test for categorical data and Mann-Whitney-Wilcoxon test for continuous data. We used the Mann-Whitney-Wilcoxon test because ASI data include many nonparametric variables and zero values [21]. The associations between continuous variables were analyzed by Spearman’s rank correlation coefficient. All analyses were performed using SPSS version 15.0 for Windows (SPSS Inc., Chicago, IL, USA).

## 3. Results

### 3.1. Characteristics of Participants

Table 1 shows the general characteristics of the two groups (*i.e.*, inmates and patients). No significant differences were found in age between the two groups. Inmates had significantly lower education levels than patients. The majority of inmates had been married and had held a full-time job during the past three years. Half of the inmates lived with a sexual partner. In contrast, less than half of the patients had ever married or had a full-time job. Only one-fifth of the patients had ever lived with a sexual partner. The inmates more frequently had a history of juvenile delinquency and had more criminal charges of illicit drug use that resulted in conviction than patients. Crimes other than illicit drug use were more common among inmates than patients. Inmates had less access to mental health service, were given less medication for psychological problems, and received less psychiatric or drug treatment than patients.

### 3.2. Severity Score and Related Items

As shown in Table 2, inmates had significantly higher composite scores (CSs) related to drug use and legal status than patients. In contrast, they had significantly lower CSs related to employment/support and psychiatric symptoms. The CSs for medical status, alcohol use, and family/social relationships did not differ between the two groups. Similar to the CSs, the interviewer severity ratings (ISRs) of the inmates were significantly higher for drug use and legal status and significantly lower for employment/support than patients. No significant differences were found for the other ISR variables between the two groups.

Table 3 shows a list of the ASI-J items that inmates and patients answered differently. With regard to employment/support status, inmates had more paid work and received more money for work in the past 30 days than patients. Inmates also received more money from illegal activities than patients. Fewer inmates received financial support compared with patients. Inmates had more people who depended on them for the majority of food, shelter, *etc*., than patients.

With regard to drug use, inmates reported significantly more days of “methamphetamine use in the past 30 days” and “multidrug use in the past 30 days” than patients. Inmates spent more money on drugs in the past 30 days than patients. No significant differences were found between the two groups in the drug-use CS items with the exception of these three items.

With regard to legal status, inmates more frequently engaged in illegal activities for profit in the past 30 days than patients. Inmates were arrested more frequently for drug charges, parole violations, and assault than patients.

With regard to psychiatric symptoms, inmates experienced fewer hallucinations and were prescribed less medication for psychological and emotional problems in the past 30 days than patients.

With regard to medical status, alcohol drinking status, and family/social relationships, no overall significant differences in CS were found between the two groups. However, significant differences were found in some of the items. For example, inmates reported significantly more days of alcohol use and spent more money on alcohol in the past 30 days than patients. Inmates also more frequently experienced serious conflicts with their family in the past 30 days than patients. Similarly, more inmates experienced serious problems with their sexual partner in the past 30 days and during their lifetime than patients.

### 3.3. Substance Use Behavior

Table 4 shows the number and percentage of participants who reported substance use/abuse lasting more than 1 year in their lifetime and who abused methamphetamine by injection. Table 4 also shows the average duration (standard deviation) of substance use/abuse of participants in prison and treatment settings. No significant difference was observed in experience of methamphetamine and alcohol abuse in their lifetime between inmates and patients. Inmates had more frequently experienced inhalant abuse and had less frequently experienced cannabis and methylenedioxymethamphetamine (MDMA) abuse than patients. Additionally, inmates had a significantly shorter duration of methamphetamine and MDMA abuse and a significantly longer duration of alcohol use than patients. Most inmates abused methamphetamine by injection (80.8%), whereas a minority of patients used the injection route (41.8%). No participants in either group reported cocaine or opiate abuse.

### 3.4. Psychiatric Symptoms

The number and percentage of participants who reported psychiatric symptoms in their lifetime are shown in Table 5. The inmates experienced less major depression, anxiety and tension, and hallucinations in their lifetime than patients. Additionally, no significant associations were found between the duration of methamphetamine abuse and these psychiatric symptoms using Spearman’s rank correlation coefficient analysis in both groups (*rs* = 0.006–0.186, *n.s.*). Moreover, inmates received less prescribed medication in their lifetime than patients. Although these psychiatric symptoms were not common among inmates, the lifetime prevalence of suicidal behavior and trouble controlling violence was not significantly lower than in patients.

### 3.5. Variables Related to Drug Abuse Severity

Similarities and differences were observed between the two groups in correlations between the drug use CS and some of the other variables. In both groups, the psychiatric status CS (inmates: *r* = 0.410, *p* < 0.05; patients: *r* = 0.283, *p* < 0.05) and the number of days that participants experienced psychological and emotional problems in the past 30 days (inmates: *r* = 0.374, *p* < 0.05; patients: *r* = 0.274, *p* < 0.05) were significantly and positively correlated with the drug use CS.

For inmates, the legal status CS (*r* = 0.328, *p* < 0.05), days of alcohol problems in the past 30 days (*r* = 0.309, *p* < 0.05), and subjective feelings of trouble with alcohol problems in the past 30 days (*r* = 0.283, *p* < 0.05) were significantly and positively correlated with the drug use CS. Additionally, the number of close friends (*r* = −0.276, *p* < 0.05) and serious problems with their children in the past 30 days (*r* = −0.366, *p* < 0.05) were significantly and negatively correlated with the drug use CS.

For patients, the employment/support status CS (*r* = 0.281, *p* < 0.05), treatment for any psychological and emotional problems in a hospital (*r* = 0.352, *p* < 0.05), and serious depression in the past 30 days (*r* = 0.383, *p* < 0.01) were significantly and positively correlated with the drug use CS.

## 4. Discussion

### 4.1. Characteristics of Methamphetamine Abusers

In the present study, significant differences were found in the backgrounds and characteristics of Stimulant Control Law inmates and participants from treatments settings. The CSs of inmates were higher for drug use and legal status and lower for employment/support and psychiatric symptoms than patients. A relatively high CS for drug status in the inmates would be attributable to their use of illegal drugs in the 30 days before arrest. Interestingly, inmates abused more frequently and spent more money on both methamphetamine and alcohol than patients. Significant correlations were observed between some items related to alcohol problems and the drug use CS in inmates. These results are consistent with the results of Russell *et al.* [22], indicating that a history of alcohol use is one of the risk factors for methamphetamine use. For Japanese inmates, methamphetamine abuse may have some association with problematic alcohol drinking.

The inmates tended to have a better employment status than patients. Most of the inmates had regular jobs, received wages, and did not receive financial support. That is, they lived a relatively financially independent life compared with patients, although many of them received a substantial amount of money illegally. Additionally, the inmates tended not to experience psychotic symptoms, such as hallucinations, and not to access medical services for their addictive problems. Many patients received treatment for the distress associated with psychotic symptoms rather than for the drug abuse itself [23]. These results indicate that inmates abused drugs within a range before their arrest, but their psychiatric symptoms were apparently not as severe as those among patients.

Problems appeared to exist in the inmates’ environments while they were young. Many of them had less education and a history of juvenile delinquency. These results are consistent with the results of Miura *et al.* [7] in which the number of admissions to a juvenile detention home significantly predicted methamphetamine use during adolescence. Moreover, a history of inhalant abuse was found in more than half of the inmates. Inhalant abuse, such as paint thinner abuse, was found to be a significant problem leading to methamphetamine abuse among young Japanese [2]. Our results, combined with these previous results, suggest that a troubled childhood may lead to illegal drug use in adulthood.

Methamphetamine-induced psychosis is reported frequently in Japanese patients diagnosed with methamphetamine dependence [6]. Additionally, Wada and Fukui [24] reported that five years of methamphetamine use is considered a turning point in terms of the occurrence of psychotic symptoms, suggesting that a shorter duration of methamphetamine abuse in inmates may be related to less frequent psychosis. However, no significant association was found between the duration of methamphetamine abuse and lifetime experience of psychosis in the inmates and patients in the present study. Interestingly, inmates reported fewer psychotic symptoms, such as hallucinations, than patients, although no significant differences were found in age between the two groups. This result may reflect the fact that inmates with acute psychosis were excluded from the study. Nonetheless, the present results suggest that inmates and patients may have different backgrounds contributing to vulnerability to methamphetamine-induced psychosis. Further studies with more inmate and patient samples using multivariate statistical analysis will be needed to reveal the factors leading to vulnerability to methamphetamine-induced psychosis in methamphetamine dependence/abuse.

With regard to route of methamphetamine administration, intravenous injection was used by most of the inmates, whereas a minority of patients used this route. These results are consistent with the results of Matsumoto *et al.* [6] in which methamphetamine-injecting subjects had more extensive criminal records than smoking subjects. Matsumoto *et al.* investigated outpatients and reported significant differences in life circumstances between injecting and smoking abusers, and injecting abusers appeared to have greater antisocial tendencies than smoking abusers. Moreover, Matsumoto *et al.* reported that although methamphetamine abusers often claimed that smoking is safer than injection, no significant differences were found in the overall occurrence of psychotic symptoms between injection and smoking. Our results consistently showed that the injection route, which was used by most of the inmates, might not be a factor causing critical psychotic problems.

The inmates were less frequently admitted to treatment facilities, possibly because of their less frequent episodes of psychosis. The inmates also expressed their psychological problems not as depression or anxiety, but rather as uncontrollable violence, suicide attempts, and interpersonal relationship problems with their families and sexual partners. These results suggest that inmates expressed their psychological problems outwardly rather than viewed their psychological problems as an inner conflict. For inmates, the correct perceptions of their problems may be especially important for treatment [25]. Treatment programs should be implemented not only for patients but also for inmates.

### 4.2. Strengths and Limitations of the Study

The present study provides basic statistical information about Japanese methamphetamine abusers in correctional settings measured by the ASI compared with abusers in treatment settings. Although Wada *et al.* [3] reported that most Japanese methamphetamine abusers belonged to correctional settings rather than treatment settings, characteristics of methamphetamine abusers in prisons have not been as well studied as those of abusers in hospital settings. The results of this study indicate many differences in the quantity and quality of methamphetamine abuse between inmates and patients. Additionally, the present study showed that the ASI could be an effective tool not only for patients but also for inmates to grasp the severity of their problems in multiple areas. The accumulation of data on incarcerated methamphetamine abusers in Japan might extend the use of the ASI as an interview tool.

One possible limitation of the present study was the sampling procedure. The participants were not recruited randomly but were limited to inmates who gave informed consent and whose doctors recognized their ability to be interviewed. Therefore, the data of this study were not obtained from methamphetamine abusers in prison as a whole, but rather only from cooperative inmates with a relatively low severity of methamphetamine dependence. Additionally, Shizuoka prison especially treated offenders who were imprisoned for the first time, and offenders mainly came from Tokyo or Shizuoka prefectures. Consequently, the results of this study reflect only a portion of the methamphetamine abuse prisoner population. Inmates and patients were also recruited at different time-points, although the situation regarding methamphetamine use in Japan minimally changed from 2002 to 2007. Moreover, the large number of statistical tests performed in the present study might make some results significant by chance. Another limitation was the relatively low sample size. A subsequent study with more subjects from other correctional facilities and multivariate statistical analysis will be necessary to confirm our conclusions.

In the future, the wide use of the ASI for methamphetamine abusers in correctional facilities will enable systematic collection of basic information from these subjects which may aid in more effective intervention. The ASI may be useful for selecting adequate treatment and re-education programs for inmates. Additionally, utilization of the ASI as a common tool among facilities that treat methamphetamine abusers, such as hospitals (or other treatment facilities), legal facilities (e.g., prisons or probation offices), and research institutes, may contribute to more effective treatment and research.

## 5. Conclusions

These findings suggest that Japanese methamphetamine abusers in correctional settings have many characteristics and environmental backgrounds that are different from abusers in medical settings. Methamphetamine abusers in correctional settings may need to have their specific problems assessed, including trouble with mental health and access to support facilities.

## Figures and Tables

**Table 1.** Characteristics of participants in each group.

	**Inmates (*n* = 52)**	**Patients (*n* = 55)**	***p* values**
Age, *M* (±SD*, range*)	38.0 (±1.5, 25–75)	35.9 (±1.2, 22–60)	*n.s.*
Years of education, *M* (±SD)	9.8 (±1.7)	11.6 (±2.3)	*p* < 0.001***
Never married, *n* (%)	9 (17.3%)	36 (65.5%)	*p* < 0.001***
Full-time employment past 3 years, *n* (%)	40 (76.9%)	24 (43.6%)	*p* < 0.001***
Lived with sexual partner past 3 years, *n* (%)	30 (57.7%)	12 (21.8%)	*p* < 0.001***
Lived with parent(s) past 3 years, *n* (%)	26 (50.0%)	24 (43.6%)	*n.s.*
Charged with juvenile delinquency, *n* (%)	42 (80.8%)	35 (63.6%)	*p* = 0.049*
Charges resulting in conviction (illicit drugs), *M* (±SD)	2.67 (±1.7)	0.98 (±1.3)	*p* < 0.001***
Charges resulting in conviction (other than illicit drugs), *n* (%)	30 (57.7%)	16 (29.1%)	*p* = 0.003**
Chronic medical problems, *n* (%)	20 (38.5%)	19 (34.5%)	*n.s.*
Medication for any psychological emotional problem, *n* (%)	16 (30.8%)	40 (72.7%)	*p* < 0.001***
History of psychiatric treatment, *n* (%)	10 (19.2%)	26 (47.3%)	*p* = 0.002**
Any drug detoxification treatments, *n* (%)	3 (5.8%)	18 (32.7%)	*p* < 0.001***
Any drug abuse treatment programs, *n* (%)	7 (13.5%)	45 (81.8%)	*p* < 0.001***
Any alcohol treatment programs, *n* (%)	2 (3.8%)	7 (12.7%)	*p* = 0.098

*n*, number of participants; *M*, mean; SD, standard deviation; *n.s.*, not significant.

*Note*: Mann-Whitney Wilcoxon test was used for the statistical comparison for Age and Years of education. The *χ^2^* test was used for the other categories.

* *p* < 0.05,

** *p* < 0.01,

*** *p* < 0.001.

**Table 2.** ASI-J composite scores (CSs) and interviewer severity ratings (ISRs).

**ASI-J area**		**Inmates (*n* = 52) Mean (SD)**	**Patients (*n* = 55) Mean (SD)**	***p* values**
Medical status	(CS)	0.06(0.02)	0.10(0.03)	*n.s.*
	(ISR)	1.04(0.25)	0.64(0.22)	*n.s.*
Employement/support	(CS)	0.45(0.04)	0.65(0.04)	p<0.001**
	(ISR)	2,85(0.43)	4.60(0.40)	p=0.002**
Alcohol use	(CS)	0.18 (0.03)	0.11 (0.02)	*n.s.*
	(ISR)	2.13 (0.38)	1.16 (0.29)	*p* = 0.086
Drug use	(CS)	0.20 (0.03)	0.14 (0.02)	*p* = 0.033*
	(ISR)	6.56 (0.24)	4.51 (0.38)	*p* < 0.001***
Legal status	(CS)	0.47 (0.02)	0.03 (0.01)	*p* < 0.001***
	(ISR)	6.98 (0.13)	0.56 (0.21)	*p* < 0.001***
Family/social relationships	(CS)	0.26 (0.03)	0.21 (0.03)	*n.s.*
	(ISR)	4.19 (0.46)	2.95 (0.30)	*p* = 0.055
Psychiatric symptoms	(CS)	0.15 (0.03)	0.24 (0.03)	*p* = 0.030*
	(ISR)	2.44 (0.44)	3.05 (0.41)	*n.s.*

*n.s.*, not significant.

*Note*: The Mann-Whitney Wilcoxon test was used for the statistical comparison.

* *p* < 0.05,

** *p* < 0.01,

*** *p* < 0.001.

**Table 3.** ASI-J items answered differently by inmates and patients.

**ASI-J items**	**Inmates (*n* = 52)**	**Patients (*n* = 55)**	***p* values**
Employment/support			
paid working days in the past 30 days, *M* (SD)	13.3 (11.95)	8.6 (11.80)	*p* < 0.05*
received money (Yen) for working in the 30 days, *M* (SD)	204,370 (248,437)	116,000 (226,830)	*p* < 0.001***
received money (Yen) for illegal activities in the 30 days, *M* (*SD*)	111,440 (426,470)	10,070 (67,285)	*p* < 0.01**
received financial support, *n* (%)	17 (65.4)	34 (18.2)	*p* < 0.001***
someone depends on you for the majority of life, *M* (SD)	0.92 (1.82)	0.37 (1.26)	*p* < 0.05*
Drug use			
days of methamphetamine use in the past 30 days, *M* (SD)	13.0 (12.28)	1.4 (5.72)	*p* < 0.001***
days of multidrug use in the past 30 days, *M* (SD)	3.6 (8.99)	1.1 (5.66)	*p* < 0.05*
money (Yen) spent on drugs in the past 30 days, *M* (SD)	72,400 (76,508)	10,560 (32,238)	*p* < 0.001***
Legal status			
days of illegal activities for profit in the past 30 days, *M* (SD)	2.8 (8.25)	0.4 (2.69)	*p* < 0.05*
experience of arrest for drug charges, *M* (SD)	2.67 (1.66)	0.98 (1.25)	*p* < 0.001***
experience of arrest for parole violations, *M* (SD)	0.44 (0.50)	0.05 (0.29)	*p* < 0.001***
experience of arrest for assault, *M* (SD)	0.48 (0.93)	0.04 (0.18)	*p* < 0.001***
Psychiatric symptoms			
experience of hallucinations in the past 30 days, *n* (%)	1 (1.9)	8 (14.5)	*p* < 0.001***
prescribed medication for psychological and emotional problems in the past 30 days, *n* (%)	10 (19.2)	32 (58.2)	*p* < 0.001***
Other status (no difference in CS)			
days of alcohol use in the past 30 days, *M* (%)	12.15 (13.01)	5.60 (10.01)	*p* < 0.001***
money (Yen) spent on alcohol in the past 30 days, *M* (SD)	34,120 (66,473)	14,180 (45,545)	*p* < 0.001***
experience of serious problems with sexual partner in the past 30 days, *n* (%)	17 (32.0)	8 (13.0)	*p* < 0.001***
experiences of serious problems with sexual partner in their lifetime, *n* (%)	41 (79.0)	30 (56.0)	*p* < 0.001***
days of serious problems with their family in the past 30 days, *M* (%)	4.37 (9.29)	0.98 (2.87)	*p* < 0.001***

*n*, number of participants; *M*, mean; SD, standard deviation

*Note*: The Mann-Whitney Wilcoxon test was used for the statistical comparison with continuous values. The *χ^2^* test was used for the other categories.

* *p* < 0.05,

** *p* < 0.01,

*** *p* < 0.001

**Table 4.** Lifetime prevalence of substance use/abuse lasting more than 1 year.

**Substance**	**Inmates (*n* = 52)**	**Patients (*n* = 55)**	***p* values**
Methamphetamine abuse, *n* (%)	50 (96.2)	52 (94.5)	*n.s.*
Duration (years), *M* (SD)	4.8 (0.63)	8.2 (0.91)	*p* = 0.001**
Drug use by injection, *n* (%)	42 (80.8)	23 (41.8)	*p* < 0.001***
Alcohol use, *n* (%)	40 (76.9)	41 (74.5)	*n.s.*
Duration (years), *M* (SD)	17.6 (1.39)	15.3 (1.48)	*p* = 0.0116*
Cannabis abuse, *n* (%)	6 (11.5)	23 (41.8)	*p* < 0.001***
Duration (years), *M* (SD)	6.5 (3.55)	7.0 (1.28)	*n.s.*
Methylenedioxymethamphetamine abuse, *n* (%)	0 (0)	6 (10.9)	*p* = 0.014*
Duration (years), *M* (SD)	0 (0)	2.3 (0.56)	*p* = 0.015*
Inhalant abuse, *n* (%)	32 (61.5)	18 (32.7)	*p* = 0.003**
Duration (years), *M* (SD)	4.1 (0.56)	3.6 (0.65)	*n.s.*

*n*, number of participants; *M*, mean; SD, standard deviation; *n.s.*, not significant

*Note*: The Mann-Whitney Wilcoxon test was used for the statistical comparison with continuous values. The *χ^2^* test was used for the other categories.

* *p* < 0.05,

** *p* < 0.01,

*** *p* < 0.001

**Table 5.** Lifetime prevalence of psychiatric symptoms.

**Symptoms**	**Inmates (*n* =52)*n* (%)**	**Patients (*n* =55)*n* (%)**	***p* values**
Serious depression (a)	9 (17.3)	24 (43.6)	*p* = 0.003**
Serious anxiety or tension (a)	14 (26.9)	25 (45.5)	*p* = 0.047*
Hallucinations (a)	3 (5.8)	22 (40.0)	*p* < 0.001***
Trouble understanding, concentrating, or remembering (a)	18 (34.6)	18 (32.7)	*n.s.*
Trouble controlling violent behavior (b)	20 (38.5)	28 (50.9)	*n.s.*
Serious suicidal thoughts (b)	22 (42.3)	29 (52.7)	*n.s.*
Suicide attempts (b)	13 (25)	18 (32.7)	*n.s.*
Prescribed medication (b)	16 (30.8)	40 (72.7)	*p* < 0.001***

*n*, number of participants; *n.s.*, not significant

*Note*: According to the ASI counting rule, (a) counts only long-lasting symptoms lasting more than three months, and (b) counts symptoms that do not last long. The *χ^2^* test was used for statistical comparisons.

* *p* < 0.05,

** *p* < 0.01,

*** *p* < 0.001
